# Identification of a Novel *MYO15A* Mutation in a Chinese Family with Autosomal Recessive Nonsyndromic Hearing Loss

**DOI:** 10.1371/journal.pone.0136306

**Published:** 2015-08-26

**Authors:** Hong Xia, Xiangjun Huang, Yi Guo, Pengzhi Hu, Guangxiang He, Xiong Deng, Hongbo Xu, Zhijian Yang, Hao Deng

**Affiliations:** 1 Center for Experimental Medicine and Department of Neurology, the Third Xiangya Hospital, Central South University, Changsha, China; 2 Department of Emergency, the Third Xiangya Hospital, Central South University, Changsha, China; 3 Department of Medical Information, Xiangya School of Medicine, Central South University, Changsha, China; 4 Department of Radiology, the Third Xiangya Hospital, Central South University, Changsha, China; 5 Department of Otolaryngology-Head Neck Surgery, the Third Xiangya Hospital, Central South University, Changsha, China; Oslo University Hospital, NORWAY

## Abstract

Autosomal recessive nonsyndromic hearing loss (ARNSHL) is a genetically heterogeneous sensorineural disorder, generally manifested with prelingual hearing loss and absence of other clinical manifestations. The aim of this study is to identify the pathogenic gene in a four-generation consanguineous Chinese family with ARNSHL. A novel homozygous variant, c.9316dupC (p.H3106Pfs*2), in the myoxin XVa gene (*MYO15A*) was identified by exome sequencing and Sanger sequencing. The homozygous *MYO15A* c.9316dupC variant co-segregated with the phenotypes in the ARNSHL family and was absent in two hundred normal controls. The variant was predicted to interfere with the formation of the Myosin XVa-whirlin-Eps8 complex at the tip of stereocilia, which is indispensable for stereocilia elongation. Our data suggest that the homozygous *MYO15A* c.9316dupC variant might be the pathogenic mutation, and exome sequencing is a powerful molecular diagnostic strategy for ARNSHL, an extremely heterogeneous disorder. Our findings extend the mutation spectrum of the *MYO15A* gene and have important implications for genetic counseling for the family.

## Introduction

Congenital or prelingual hearing loss is a common sensorineural disorder, with a prevalence of about one in 500–1,000 at birth, and at least half of the cases are caused by genetic factors [1,2]. At least 70% of the cases manifest with isolated hearing loss without other associated clinical features, which is classified as nonsyndromic deafness [3]. Hereditary hearing loss mainly displays autosomal recessive or autosomal dominant transmission [2], and X-linked [4] or mitochondrial inheritance [5] is occasionally reported. Most of hereditary deafness manifests as autosomal recessive nonsyndromic hearing loss (ARNSHL) [3].

ARNSHL is an extremely heterogeneous disease, generally manifested with congenital or prelingual hearing loss without associated clinical symptoms, though postlingual hearing loss has also been reported [2,6]. The individuals with early-onset deafness often encounter obstacles for linguistic development [7]. Since identification of the gap junction protein beta-2 gene (*GJB2*) as the disease gene for ARNSHL [8], more than 42 genes have been identified and at least 1,949 pathogenic variants have been reported [9]. Mutations in these genes affect cochlear homeostasis, cellular organization, neuronal transmission, cell growth, differentiation and survival, and tectorial membrane associated proteins [2]. Cochlear implantation has been reported to offer satisfactory auditory performance to patients with severe to profound deafness caused by mutations in the *GJB2* gene, the solute carrier family 26 member 4 gene (*SLC26A4*), the otoferlin gene (*OTOF*) [10], or the myosin XVa gene (*MYO15A*) [11]. Genetic diagnosis plays an important role in prognosis evaluation, clinical management, and prenatal diagnosis for ARNSHL families [12].

It is difficult to identify causative mutations using regular Sanger sequencing because of high heterogeneity of ARNSHL. Recently, exome sequencing has been introduced and confirmed as an effective alternative strategy [13]. In this study, a novel homozygous mutation in the *MYO15A* gene was identified in a Chinese ARNSHL family by exome sequencing.

## Materials and Methods

### Subjects

A four-generation consanguineous Chinese Han family with ARNSHL was recruited, and four members of the family participated in this study. Bilateral prelingual deafness was observed in the two siblings (IV:1 and IV:2, Fig 1A), who received neither hearing aids nor cochlear implantation in their childhood. However, their parents (III:1 and III:2, Fig 1A) had normal hearing. Two hundred ethnically-matched unrelated subjects (age 29.5±6.5 years) with normal hearing were enrolled as controls. Clinical and audiometric assessments were performed, and peripheral blood samples were collected from all the subjects after obtaining written informed consent from the participants or guardians. The study was approved by the Institutional Review Board of the Third Xiangya Hospital, Central South University, China.

**Fig 1:**
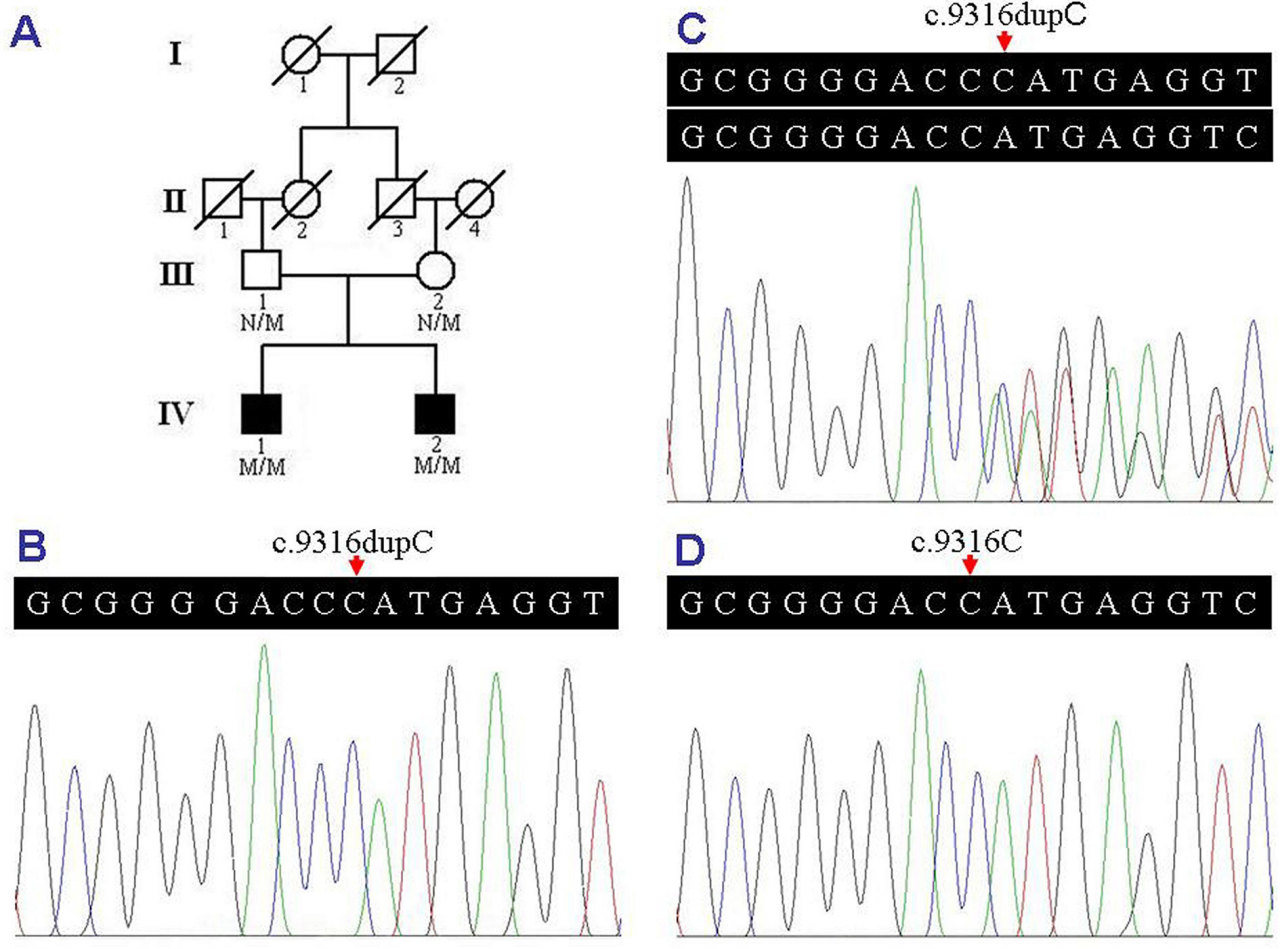
Pedigree and sequence analysis of an ARNSHL family. (A) Pedigree of the ARNSHL family. N, normal; M, the *MYO15A* c.9316dupC variant. (B) The homozygous *MYO15A* c.9316dupC variant of the affected individual (IV:2). (C) The heterozygous *MYO15A* c.9316dupC variant of the unaffected individual (III:1). (D) The *MYO15A* gene sequence of a normal control. ARNSHL, autosomal recessive nonsyndromic hearing loss; *MYO15A*, the myosin XVa gene.

### Clinical evaluations

Clinical and audiometric assessments were performed on the subjects of the family in the Third Xiangya Hospital, Changsha. Pure tone audiometry (PTA), tympanometry, acoustic reflex (AR) thresholds, auditory brainstem responses (ABR), transient evoked otoacoustic emission (TEOAE) and distortion product otoacoustic emission (DPOAE) were conducted. Magnetic resonance imaging (MRI) was carried out to exclude congenital inner ear malformations. The hearing level was assessed at 250, 500, 1000, 2000, 4000 and 8000 Hz by PTA, and sorted into normal (<20 dBHL), mild (20–40 dBHL), moderate (41–70 dBHL), severe (71–95 dBHL), and profound (>95 dBHL) deafness [14].

### Whole exome sequencing and variant analysis

Genomic DNA was extracted from peripheral blood samples of all the subjects using standard phenol-chloroform extraction method [15]. Exome sequencing was conducted by Novogene Bioinformatics Institute, Beijing, China. At least 1.5 micrograms (μg) of genomic DNA from the proband (IV:2, Fig 1A) was sheared by Covaris sonicators, and was enriched, hybridized, and captured on the Agilent SureSelect Human All Exon V5, following the manufacturers’ procedures. The captured library was sequenced with the Illumina HiSeq 2000 sequencing instruments. The average sequencing depth of 57.36× provided enough depth to exactly call variants at 97.4% of targeted exome [16].

The clean reads without adapter or debased reads were mapped to the human reference genome (UCSC hg19, http://genome.ucsc.edu/) using Burrows-Wheeler Alignment tool (BWA) [17,18]. Single nucleotide polymorphisms (SNPs) and insertions/deletions were identified by the Sequence Alignment/Map tools (SAMtools) [19], and then Picard was applied to mark duplicate reads. All variants were screened with the SNP database version 142 (dbSNP142), 1000 Genomes Project (version 2014 October), and NHLBI Exome Sequencing Project (ESP) 6500. Functional prediction was carried out by Sorting Intolerant from Tolerant (SIFT) and Polymorphism Phenotyping version 2 (PolyPhen-2). Candidate variants were annotated by the ANNOVAR (Annotate Variation) software [20].

### Direct Sanger sequencing and functional prediction

Direct Sanger sequencing was performed to confirm potential causative variants in the family with ABI3500 sequencer (Applied Biosystems, Foster City, CA, USA) [21]. Primer sequences for pathogenic variant in the *MYO15A* gene (NM_016239.3) were designed as follows: 5′-TGCCCACCCTGTTCTTATGT -3′ and 5′-ACTCACTGCTTGGAGCTGGT-3′. MutationTaster was applied to the functional prediction of the *MYO15A* pathogenic variant [22].

## Results

### Clinical findings

Both patients (IV:1 and IV:2, Fig 1A) presented with deafness and dumbness. Bilateral profound sensorineural hearing loss with thresholds over 95 dBHL was revealed by PTA. Type A tympanometric curve was shown by acoustic immitance measurement, and no inner ear anomaly was discovered by MRI in the two patients. The ABR at 97 dB, AR, TEOAE and DPOAE were absent in both ears of the proband (IV:2, Fig 1A) and the right ear of the elder sibling (IV:1, Fig 1A), while the waves I, III and V of ABR were elicited at 80 dB, remarkably elevated acoustic reflex threshold (80–105 dB) was recorded, and low amplitude DPOAE was elicited at 500, 1000 and 4000 Hz in the left ear of the IV:1 patient, which suggested that some residual hearing might exist in the left ear of the IV:1 patient. The clinical information of the ARNSHL family was summarized in Table 1.

**Table 1:** Phenotypes and genotypes of the ARNSHL family.

Subjects	Age	Hearing loss	DPOAE	ABR	AR	MRI	*MYO15A* c.9316dupC mutation
**III:1**	58 y	Normal	Bil (+)	Bil (+)	Bil (+)	Normal	Heterozygous
**III:2**	57 y	Normal	Bil (+)	Bil (+)	Bil (+)	Normal	Heterozygous
**IV:1**	32 y	Bil profound	L (A), R (-)	L (A), R (-)	L (A), R (-)	Normal	Homozygous
**IV:2**	28 y	Bil profound	Bil (-)	Bil (-)	Bil (-)	Normal	Homozygous

A, abnormality; ABR, auditory brainstem responses; AR, acoustic reflex; Bil, bilateral; DPOAE, distortion product otoacoustic emissions; L, left; MRI, magnetic resonance imaging; *MYO15A*, the myosin XVa gene; R, right; y, years; +, presence;-, absence

### Exome sequencing

A total of 19,816,364 pairs of sequenced reads with the average read length of 125 bp were generated by exome sequencing, and 98.76% (19,569,878) of sequenced reads passed the quality assessment and were mapped to 99.81% of the human reference genome [16]. Known variants identified in dbSNP142 with minor allele frequency (MAF) >1%, 1000 Genomes Project with a frequency of >0.5%, and NHLBI ESP6500 were filtered out. PolyPhen-2 and SIFT were applied to predict functional effects of non-synonymous SNPs. Subsequently, a homozygous *MYO15A* c.9316dupC variant was observed in the proband (IV:2, Fig 1A and 1B) and other possible pathogenic mutations for ARNSHL were excluded.

### Identification of pathogenic mutation

The homozygous *MYO15A* c.9316dupC variant was confirmed by Sanger sequencing. The same homozygous *MYO15A* variant was also detected in his affected sibling (IV:1, Fig 1A), and the heterozygous *MYO15A* c.9316dupC variant was identified in both of his unaffected parents (III:1 and III:2, Fig 1A and 1C). However, the variant was absent in two hundred ethnically-matched unrelated controls (Fig 1D). The homozygous *MYO15A* c.9316dupC variant, which co-segregated with the phenotype of deafness and dumbness in the family, and was predicted to lead to a shift in the reading frame at amino acid position 3106 and a premature stop codon (p.H3106Pfs*2) by MutationTaster [22], might be the disease-causing mutation in the ARNSHL family.

## Discussion

In 1995, a disease gene locus (deafness, autosomal recessive 3; *DFNB3*) for ARNSHL was first mapped to chromosome 17p-17q12 by linkage analysis of two large multi-generation families from Bengkala, Bali [23], and then was further refined to chromosome 17p11.2 [24]. In 1998, the homozygous p.N2111Y, p.I2113F and p.K2601* (previously known as p.N890Y, p.I892F and p.K1300*) mutations in the *MYO15A* gene were identified in three unrelated DFNB3 families [25,26]. A hemizygous p.T2205I mutation of the *MYO15A* gene was also reported to be associated with moderately severe hearing loss in a Smith-Magenis syndrome (del(17)p11.2) patient [27].

**Fig 2:**
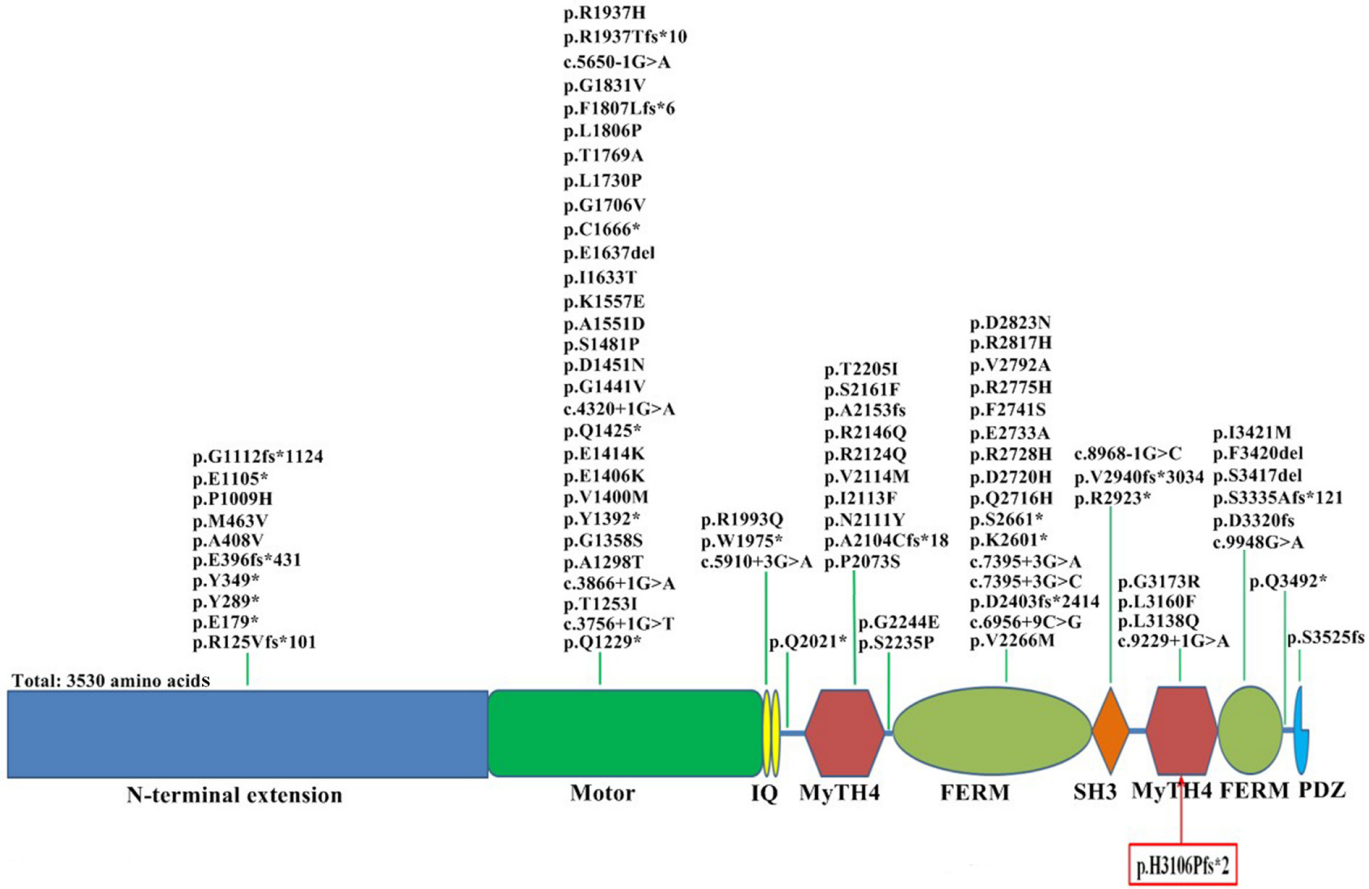
The schematic structure and the mutations of the human myosin XVa. The myosin XVa consists of 3530 amino acids, including an N-terminal extension domain and Motor domain, two light chain binding IQ motifs, two myosin-tail homology 4 (MyTH4) domains and band 4.1/ezrin/radixin/moesin (FERM) domains, a Src-homology-3 (SH3) domain and a C-terminal class I PDZ-ligand domain. The novel *MYO15A* mutation in this study is showed with red box at the bottom of the figure, and previously reported mutations are displayed at the top of the figure. *MYO15A*, the myosin XVa gene.

Homozygous *MYO15A* mutations cause 6.2% of ARNSHL in Turkey [3], and mutations in the *MYO15A* gene account for no less than 5% of autosomal recessive profound hearing loss in Pakistan [27]. To date, at least 86 pathogenic variants of the *MYO15A* gene have been reported in deafness populations [3,11,26–47], which are summarized in Fig 2. The p.D2720H mutation in the *MYO15A* gene is considered as a founder mutation in Pakistan [46], and the p.R1937Tfs*10 and p.S3335Afs*121 mutations in the *MYO15A* gene were also identified as founder mutations in Turkish population [3]. Most mutations in the *MYO15A* gene are connected with congenital severe to profound sensorineural deafness [27,46], while some patients also display progressive hearing loss [11,36]. Intriguingly, a homozygous p.Y289* mutation in the *MYO15A* gene was associated with maintenance of considerable residual hearing in two Turkish patients [3]. High frequency hearing loss or retention of some hearing at low frequency was also reported in patients with *MYO15A* mutations [11,46].

The *MYO15A* gene contains 66 exons and encodes several alternatively spliced transcripts in the inner ear [25]. The complete transcript consists of 3530 amino acids, including a long N-terminal extension encoded by exon 2, an N-terminal motor domain, two light chain binding IQ motifs, and a tail region containing two myosin-tail homology 4 (MyTH4) domains, two band 4.1/ezrin/radixin/moesin (FERM) domains, a Src-homology-3 (SH3) domain and a C-terminal class I PDZ-ligand domain [25,48]. Myosin XVa protein is mainly expressed in the cuticular plate and stereocilia of the cochlear inner and outer hair cells [25], and is commonly localized at the tips of inner ear sensory cell stereocilia [49]. Myosin XVa is involved in staircase formation of the hair bundle, which is indispensable to sound detecting and head movement [48,49].

Homozygous p.C1779Y mutation in the *Myo15* gene, a murine homologue of the human *MYO15A* gene, cause profound sensorineural deafness, vestibular defects, and extremely short stereocilia on the inner and outer hair cells in *shaker-2* mice [50].

In this study, the homozygous c.9316dupC variant in the *MYO15A* gene was identified in the two affected siblings, but was absent in the unaffected parents and two hundred normal controls. The homozygous c.9316dupC variant in the *MYO15A* gene co-segregated with the phenotype of deafness in the ARNSHL family and might be the disease-causing mutation.

Both affected siblings display bilateral prelingual, profound sensorineural hearing loss, in accordance with most *MYO15A*-associated ARNSHL phenotypes [3]. Their language acquisitions were hindered by profound prelingual deafness [7], thus they also present with dumbness phenotypes. The audiometric tests of the IV:1 patient implied that the patient might have some residual hearing, consistent with the previous report [46].

The novel c.9316dupC variant in the *MYO15A* gene, located in the second MyTH4 domain [43], was predicted to result in a shift in the reading frame and a premature stop codon (p.H3106Pfs*2) by MutationTaster [22], which leads to a truncated protein missing part of the second MyTH4 domain, the second FERM domain and PDZ-ligand in the tail region of myosin XVa (Fig 2). More than ten mutations have been reported in the second MyTH4 and FERM domain of myosin XVa (summarized in Fig 2). The MyTH4-FERM region is involved in formation of the Myosin XVa-whirlin-Eps8 complex [51] and microtubule binding [52]. Thus, the c.9316dupC variant in the *MYO15A* gene might interfere with formation of the Myosin XVa-whirlin-Eps8 complex, which is indispensable for stereocilia elongation and sound detecting [48,51].

## Conclusion

The homozygous c.9316dupC variant in the *MYO15A* gene was the pathogenic mutation in our ARNSHL family. Our study demonstrated that exome sequencing is a powerful molecular diagnostic strategy for ARNSHL, an extremely heterogeneous genetic disorder. Our findings extend the mutation spectrum of the *MYO15A* gene, and have implication in genetic counseling for the ARNSHL family.
